# Oxygen and nutrient delivery in tissue engineering: Approaches to graft vascularization

**DOI:** 10.1002/term.2932

**Published:** 2019-07-30

**Authors:** Timo Rademakers, Judith M. Horvath, Clemens A. van Blitterswijk, Vanessa L.S. LaPointe

**Affiliations:** ^1^ Department of Instructive Biomaterials Engineering, MERLN Institute for Technology‐Inspired Regenerative Medicine Maastricht University Maastricht The Netherlands; ^2^ Complex Tissue Regeneration, MERLN Institute for Technology‐Inspired Regenerative Medicine Maastricht University Maastricht The Netherlands

**Keywords:** regenerative medicine, tissue engineering, vascularization

## Abstract

The field of tissue engineering is making great strides in developing replacement tissue grafts for clinical use, marked by the rapid development of novel biomaterials, their improved integration with cells, better‐directed growth and differentiation of cells, and improved three‐dimensional tissue mass culturing. One major obstacle that remains, however, is the lack of graft vascularization, which in turn renders many grafts to fail upon clinical application. With that, graft vascularization has turned into one of the holy grails of tissue engineering, and for the majority of tissues, it will be imperative to achieve adequate vascularization if tissue graft implantation is to succeed. Many different approaches have been developed to induce or augment graft vascularization, both *in vitro* and *in vivo*. In this review, we highlight the importance of vascularization in tissue engineering and outline various approaches inspired by both biology and engineering to achieve and augment graft vascularization.

## 1 INTRODUCTION

The aim of regenerative medicine is the repair and regeneration of damaged tissues and failing organs by combining knowledge from the fields of cell biology, material science, engineering, and medicine. Although great strides have been made in the past decades producing clinical trials involving tissue‐engineered constructs, the field still faces some serious challenges. One of these challenges is the inability to upscale *in vitro* tissue constructs to achieve clinically relevant tissue masses. Indeed, a lack of oxygen and nutrient supply constrains the size of *in vitro* engineered tissue (Griffith & Naughton, 2002). Under normal physiological conditions, tissues rely on the body's circulatory system to supply individual cells with nutrients and oxygen for their survival, thus making vascularization a vital prerequisite for successful tissue (re‐)generation (Novosel, Kleinhans, & Kluger, 2011). In this review, we will recapitulate the importance of vascularization in tissue engineering and discuss both *in vitro* and in vivo biologically inspired methods, as well as engineering approaches to achieve and augment vascularization in tissue‐engineered constructs.

## 2 IMPORTANCE OF VASCULARIZATION IN PHYSIOLOGY AND TISSUE ENGINEERING

Supplying oxygen to tissue is one of the major functions of the circulatory system, aiding cells in maintaining cellular respiration. Oxygen is supplied via passive diffusion, resulting in an oxygen gradient that declines from a tissue's periphery towards its core. This effectively introduces an upper physical limit for avascular tissue size (Folkman & Hochberg, 1973; Pittman, 2011), and it is due to the intricate network of arteries, veins, and capillaries that native tissue size is not restricted to the diffusion limit of oxygen, which was first studied by August Krogh (1919), and is limited to roughly 100–200 μm. Although the body has developed ways to supply all its cells with sufficient oxygen, the implantation of tissue‐engineered constructs creates a challenge, as host‐mediated angiogenesis is a slow process. Previous research has shown that it requires approximately 1–2 weeks for complete vascularization of a 3‐mm tissue construct upon implantation, a period during which cells inside the construct are not (adequately) supplied with nutrients and experience a lack of oxygen (Farris, Rindone, & Grayson, 2016).

### 2.1 Oxygen supply, hypoxia, and hyperoxia

In particular, cells located deep inside the tissue construct will experience a hypoxic environment (Samuel & Franklin, 2008), a condition that contributes to apoptosis after prolonged exposure *in vivo* by inducing stress‐related pathways because oxidative phosphorylation is no longer possible and anaerobic respiration is insufficient to meet the metabolic demand. Mesenchymal stem cells (MSCs), for example, have been shown to be resilient to transient hypoxia for up to 48 hr through anaerobic metabolic pathways, yet mitochondrial stress leading to increased caspase‐3 activation, together with the build‐up of lactic acid and the resulting low pH, will eventually induce apoptosis (Das, Jahr, van Osch, et al., 2009; Potier et al., 2007).

Attempts to increase local oxygen tension to alleviate tissue hypoxia by exposure to an excess of oxygen, a condition referred to as hyperoxia, may equally cause cell damage due to the production of reactive oxygen species (Floyd, 1990; Mach, Thimmesch, Pierce, & Pierce, 2011). Importantly, hyperoxic conditions are not only detrimental *in vivo*, but may also interfere with cell growth in cultures, slowing proliferation and eventually leading to apoptosis (Cacciuttolo, Trinh, Lumpkin, & Rao, 1993). The importance of oxygen in tissue engineering and its paradoxical behaviour herein have recently been reviewed (Sthijns, van Blitterswijk, & LaPointe, 2018), and thus lie beyond the scope of this review. Yet achieving a balanced oxygen supply is crucial for successful tissue engineering approaches and should be considered carefully when developing new tissue engineering constructs.

### 2.2 Maintaining homeostasis—Nutrient supply and waste removal

Cellular functions such as proliferation and differentiation are enabled through the supply of essential nutrients and the removal of metabolic waste products and toxins, thereby maintaining cellular homeostasis (Pugsley & Tabrizchi, 2000). In tissue constructs *in vitro*, cells in the core of a scaffold may suffer both from nutrient deprivation and waste product build‐up as cells closer to the periphery will absorb the majority of the nutrients from the medium (Fisher, 2007). Similarly, a lack of vascularization will result in the same issues *in vivo*. Stem cells in particular heavily rely on nutrients to regulate their transition between a quiescent and activated state, as entry into the cell cycle requires high amounts of energy (Cavallucci, Fidaleo, & Pani, 2016). As such, not only can a lack of nutrients cause starvation, but it can also influence behaviour such as proliferation and differentiation.

### 2.3 Paracrine signalling and immune functions

Signal transduction and cellular communication through paracrine signalling are important physiological aspects that are partly mediated by the vasculature. Differentiation, adhesion, tissue repair, and a plethora of other cellular functions are the result of such signalling, allowing cells to correctly respond to their micro‐environment, as has been more extensively reviewed previously (Augustin & Koh, 2017; Orr & Eberhart, 2015). Although not often considered in tissue engineering, it is an additional aspect to be taken into account in tissue construct vascularization.

In contrast, the role of vascularization in the immune system is more easily recognized. The immune system requires the circulation of blood (and lymph) for antigen recognition and immune cell activation. The cardiovascular system ensures both that potential pathogens are brought into contact with immune cells of the primary and secondary immune response and that activated cells are capable of reaching sites of infection. This function has special pertinence to tissue grafts after implantation. On the one hand, an immune response encompasses potential detrimental effects in which the construct may be recognized as foreign and elicit an immune response, but on the other hand, cells of the immune system can also be instrumental in local tissue remodelling (Kwee & Mooney, 2015). For example, upon establishing vascularization, monocytes or macrophages aided neovascularization by enhancing vessel stability in engineered constructs (West, Sefton, & Sefton, 2018). This shows that paracrine and immune functions are an important facet of vascularization that have implications for vascularization strategies.

### 2.4 Importance to patients post‐implantation

Although all these aspects of vascularization are important in both the short and long term, vascularization of tissue constructs primarily prevents their necrosis shortly after implantation. Especially in large‐scale defects, the use of grafts previously often failed, and even though allografts or synthetic bone grafts do exist, the golden standard still are autologous vascularized tissue grafts (Sen & Miclau, 2007; Zimmermann & Moghaddam, 2011). Although the use of these vascularized grafts has improved the effectiveness of treating these large defects, and it reduces post‐operative infection risk, as shown by a recent meta‐analysis (Azi et al., 2016), this approach presents an important disadvantage. Acquiring the tissue requires additional surgical procedures and creates significant donor site morbidities, accompanied by, for example, reduced function of the donor site, additional scarring, and increased pain for the patient (Betz, 2002). A recent study in rabbits showed how the use of a vascularized periosteal flap in combination with a bioglass‐β‐TCP monoblock and BMP‐2 supplementation was effective in repairing a large femoral defect (Pan et al., 2018), emphasizing that indeed prevascularization strategies are effective in preclinical models of critical size defects.

Yet, to date, it is therefore mostly relatively thin *in vitro* engineered tissues such as skin or cartilage that can be successfully transplanted without the risk of hypoxia and eventual necrosis (Novosel et al., 2011). These tissues can effectively be supplied with nutrients and oxygen by diffusion from blood vessels located further away, or are avascular, and are thus less reliant on neovascularization. Yet, in other tissue grafts, vascularization will be of key importance to achieve successful transplantation.

Given the variety of functions of the vasculature, it is understandable that vascularizing tissue‐engineered grafts has proven challenging. In an effort to introduce effective neovascularization, multiple concepts have been designed and experimentally tested, resulting in a variety of different approaches to solving the problem of vascularization.

## 3 ENHANCING ANGIOGENESIS TO ACHIEVE VASCULARIZATION IN TISSUE ENGINEERING

In general, the formation of new blood vessels, that is, neoangiogenesis, may be subdivided into three processes: vasculogenesis, angiogenesis, and arteriogenesis (Stavrou, 2008). Vasculogenesis involves the differentiation of angioblasts, mesodermal progenitor cells, and subsequent *de novo* formation of blood vessels. Although traditionally considered restricted to *in utero* development, some studies have demonstrated the possibility of adult vasculogenesis from circulating endothelial progenitor cells (Aicher, Rentsch, K‐i, et al., 2007; Asahara et al., 1999; Ribatti, Vacca, Nico, Roncali, & Dammacco, 2001). Angiogenesis, which is defined as the sprouting of new capillaries (<10–15 μm in diameter) from pre‐existing vasculature, however, usually occurs without a significant contribution of progenitor cells. In clinical literature, the term angiogenesis is used more liberally, also referring to the sprouting of larger vessels (>1–2 mm in diameter). Arteriogenesis, which describes shear stress‐induced remodelling of the vasculature, is used in most other literature to characterize the formation of larger arteries (Kovacic et al., 2008; Risau, 1997). Although various nuances of blood vessel formation still remain to be fully understood, previous research has provided a strong basis for tissue engineering and regenerative medicine to build upon in the effort to produce vascularized tissue constructs.

### 3.1 Prevascularization strategies

Although perfusion bioreactors have been able to increase mass transport inside three‐dimensional (3D) tissue constructs to form larger, more uniform tissue grafts *in vitro*, their merit upon implantation *in vivo* is at current not evident (Gaspar, Gomide, & Monteiro, 2012; Janssen, Oostra, Av, et al., 2006; Radisic, Marsano, Maidhof, Wang, & Vunjak‐Novakovic, 2008). As such, the engineered tissue graft effectively has to rely again on natural neovascularization from the recipient. Recognizing this shortcoming, prevascularization efforts have tried to ensure the development of a capillary network within the tissue construct prior to transplantation *in vivo*. Using such an approach would require only inosculation, which is the fusing of the host blood vessels with the engineered vasculature post‐implantation. This would thereby reduce the time needed to vascularize the implant, consequently avoiding avascular periods and reducing the duration of hypoxia (Mitchell & Morrison, 2017).

Two important aspects of prevascularization that warrant more discussion, independent of the approach chosen, is the type of endothelial cells and their ratio to tissue‐specific (stem) cells. Although endothelial cells do assemble rapidly into a vascular‐like structure after seeding on an appropriate matrix, using patient‐derived endothelial cells has proven challenging, as harvesting a sufficient number from a patient is difficult, and these isolated cells have little active proliferation in culture (Black, Berthod, L'heureux, Germain, & Auger, 1998; Boyer et al., 2000; Unger, Sartoris, Peters, et al., 2007). Moreover, endothelial cells from different organs show distinct phenotypes *in vivo* (Rafii, Butler, & Ding, 2016), and the application of an arbitrary endothelial cell type may not be sufficient. Endothelial progenitor cells (EPCs) have been used as an alternative (Duttenhoefer, Lara de Freitas, Meury, et al., 2013), and have the benefit of being easily harvested from the bone marrow or peripheral blood, and can also be rapidly expanded in culture (Wu et al., 2004). Details on applications and challenges of Endothelial Progenitor Cell (EPC) in regenerative medicine have been reviewed previously (Chong, Ng, & Chan, 2016).

In order to induce a more effective vascularization *in vitro*, various studies have also used additional (mural) cell types in conjunction with endothelial cells. Mural precursor cells, for instance, have been shown to play a role in vascular remodelling, and cocultures including them have shown better‐developed vascular structures in culture (Bodnar, Rodgers, Chen, & Wells, 2013; Kim et al., 2015; Koike et al., 2004). MSCs in particular have shown a great potential in aiding vascularization in regenerative medicine, most likely by a combination of their immunomodulatory effects and their paracrine release of growth factors, such as vascular endothelial growth factor (VEGF; Andrzejewska, Lukomska, & Janowski, n.d.; Shafiee et al., 2017). This enhanced vascularization has been shown effective, for example, in hydrogels for wound healing (Alapure et al., 2018), in vascular flaps (Linard et al., 2018), periosteal flaps (Nau et al., 2017), and bone scaffolds (Rottensteiner‐Brandl et al., 2018).

Beyond the cell type, selecting an appropriate ratio of endothelial cells within the graft is also critical. The ratio of vascular cells to tissue‐specific cells in a tissue construct has been investigated and confirmed the notion that using too many endothelial cells actually decreases graft neovascularization, likely due to a higher metabolic load and increased competition for nutrients (Paik et al., 2015). At current, no unified accepted ratio has been reported, partially at depends greatly on tissue type and graft size.

### 3.2 *In vitro* prevascularization strategies

As alluded to by its name, *in vitro* prevascularization focuses on vascularization of tissue constructs prior to implantation. These techniques mainly refer to seeding scaffolds not only with tissue‐specific cells but also with endothelial cells to allow them to naturally form prevascular networks within the tissue construct.

**Figure 1:**
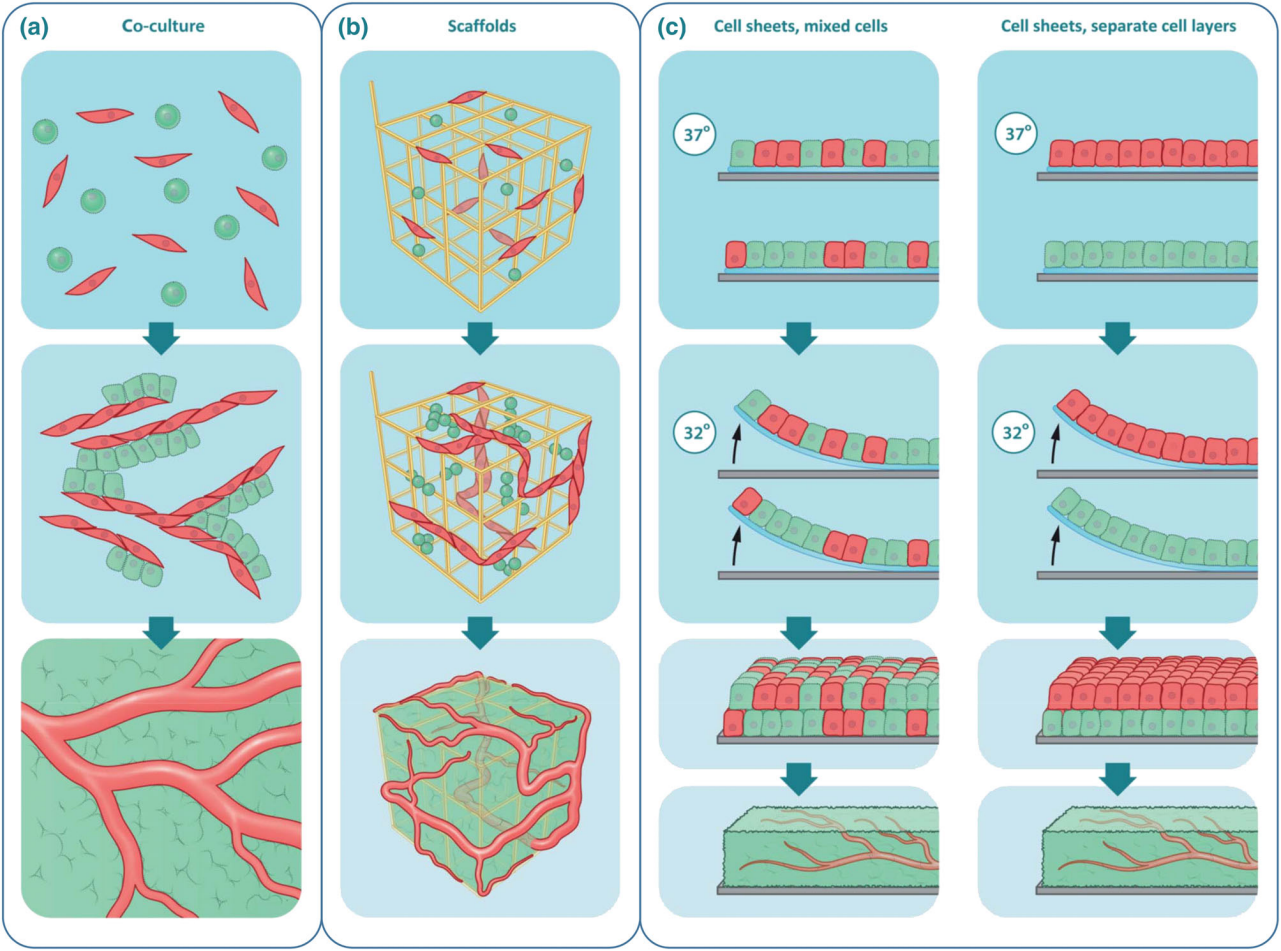
*In vitro* prevascularization approaches in tissue engineering. (a) One of the most straightforward approaches to *in vitro* prevascularization is the coculture of tissue‐specific, progenitor or stem cells (green) and endothelial cells (red). Such coculture systems can lead to the formation of vascular structures, which accelerate graft perfusion upon implantation. Although vascular structure formation has been shown, this approach is limited by the fact that it is mostly employed in 2D culture environments and by the fact that the vessels are not functional. (b) A similar approach is the use of a scaffold, which can be moulded to the needs of the graft. Moreover, the scaffold can help to guide vascularization into an intricate 3D vascular network. (c) A newer method that attempts to construct 3D vascular structures without the use of a scaffold is the use of cell sheets. By using thermoresponsive sheets, sheets of cells can be created and stacked on top of each other, to build a 3D structure layer by layer. An advantage of this system is that the native extracellular matrix is preserved. Yet this approach is also limited in the number of layers, that is, the size of the tissue graft that can be constructed [Colour figure can be viewed at http://wileyonlinelibrary.com]

A first means of establishing prevascularization is the use of coculture systems (Figure 1a). Previous studies have shown that embedding endothelial cells in tissue‐engineered constructs does indeed result in capillary structures with a branching morphology similar to that in the human body, and that these structures are capable of long‐term survival *in vitro* (Black et al., 1998) and *in vivo* (Chen, Thein‐Han, Weir, Chen, & Xu, 2014; Koike et al., 2004). In addition, studies in mice demonstrated that such prevascularized tissue grafts markedly accelerated complete vascularization post implantation compared with nonendothelialized skin grafts (Tremblay, Hudon, Berthod, Germain, & Auger, 2005). In this specific study, the complete tissue graft was perfused just 4 days after transplantation, indicating a connection to the host circulation, whereas the nonendothelialized skin graft showed similar results only after 2 weeks. These results support the notion that prevascularized tissue grafts require only inosculation rather than complete neovascularization, and as a result face a shorter period of hypoxia and nutrient deprivation post‐transplantation and thus have a higher survival *in vivo* (Levenberg et al., 2005; Nör et al., 2001). Notably, inosculation is a process that may remain independent of graft tissue size and thus appears to be a promising technique to overcome tissue graft necrosis *in vivo* for various tissue types (Laschke, Vollmar, & Menger, 2009).

As not all tissue‐engineered constructs consist of only (cocultured) cells, prevascularization strategies may also use a scaffold as a template for tissue formation (Figure 1b). Biomaterials for scaffold fabrication include ceramics (e.g., for bone regeneration), synthetic polymers, or natural polymers, which have all been reviewed elsewhere (Bassas‐Galia, Follonier, Pusnik, et al., 2017; Edgar et al., 2016; Moroni, Nandakumar, de Groot, van Blitterswijk, & Habibovic, 2015). Another method to obtain a 3D scaffold is tissue decellularization, a process in which all cells are removed from a donor tissue, after which (stem) cells are reimplanted into the remaining matrix. Independent of their origin, these scaffolds are meant to provide a bioactive environment that may, to a certain extent, mimic the extracellular matrix (ECM), and thereby facilitate cell adhesion and activate signalling pathways for proliferation and (directed) differentiation. Cell‐type specific responses due to the characteristics of the scaffold material, for example, substrate stiffness and bioactive groups, have been reported (Rothdiener et al., 2016; Seeger et al., 2015). Careful selection of such scaffolds is required in order to not interfere with vascularization *in vitro*.

To forego the use of a scaffold, cell sheets may be used for *in vitro* prevascularization (Figure 1c). In this approach, the sheets are stacked to achieve a tissue graft. This concept is based on the principles described in developmental biology, in which every organ essentially originates from sheets of endoderm, mesoderm, and ectoderm (Ray & Niswander, 2012). The method often relies on monocultures of tissue‐specific cells cultured on a temperature‐responsive polymer, such as poly‐*N*‐isopropylacrylamide, that allows cells to adhere and proliferate at 37°C, but enables their trypsin‐free harvest upon lowering the temperature, thereby resulting in a single cell sheet with intact cell–cell junctions and an established ECM. Multiple sheets may be stacked to obtain thicker tissue grafts (Labbé, Marceau‐Fortier, & Fradette, 2011; Yamato & Okano, 2004). Importantly, they may also be vascularized via coculture with endothelial cells or by including endothelial cell sheets in a multilayered construct (Asakawa et al., 2010; Sekiya, Shimizu, Yamato, Kikuchi, & Okano, 2006). Nevertheless, increasing the size of the tissue graft may result in tissue necrosis, which effectively limits the number of sheets that can be stacked as the diffusion distance of oxygen and nutrients is still limited, and the endothelial cell sheets may not form blood vessels. One possibility to overcome this is to combine cell sheet technology with perfusion bioreactors, allowing for the vascular integration of newly stacked cell sheets by ensuring the flow of medium. Currently, there remains an upper limit of 12 layers, thereby limiting graft size (Sakaguchi et al., 2013; Sakaguchi, Shimizu, & Okano, 2015). Still this technology holds potential as it allows for a combination of prevascularization with the added benefit of transplanting the established cellular environment, which has positive effects on continued neovascularization post‐implantation (Sekiya et al., 2006).

### 3.3 *In vivo* prevascularization

**Figure 2:**
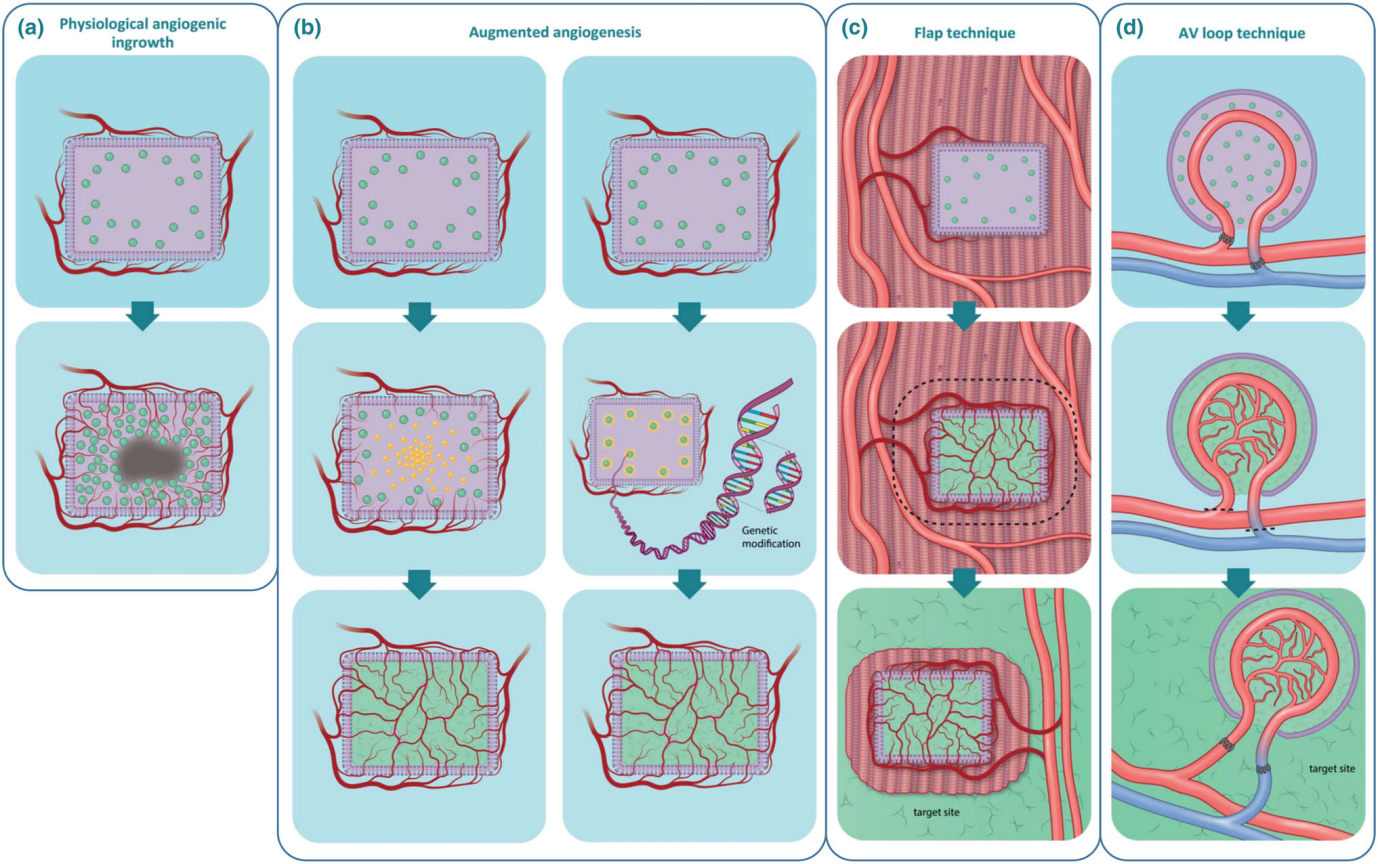
*In vivo* prevascularization approaches in tissue engineering. (a) The major issue of relying on physiological angiogenic ingrowth into a (nonprevascularized) tissue graft is the lack of adequate and timely vascularization, leading to cell necrosis in the core of the graft. (b) A first approach to tackle this issue *in vivo* is by enhancing the naturally occurring angiogenic response within the graft, either by loading the graft/scaffold with (a gradient of) proangiogenic factors (left panel) or by genetic modification of the graft cells to induce enhanced secretion of angiogenic factors (right panel). Both strategies are aimed at enhancing angiogenesis directly at the target site. Two alternative approaches use an ectopic implantation site prior to the actual target site to establish a vascular network. (c) The flap technique is one of these two techniques and is characterized by preimplantation in muscle tissue to prevascularize the graft. Upon transplantation, however, part of the muscle tissue will also be transplanted, and as such will cause tissue damage at the ectopic implantation site. (d) An improved technique that has been applied is the arteriovenous (AV) loop technique. It follows the same principle as the flap technique, yet can be established at any location, and without causing damage of the ectopic implantation site. The principle is based on an encapsulated graft, which incorporates an AV loop to facilitate initial perfusion until further vascularization can be established. The vascularized graft then can be transplanted to the target site. Similar approaches such as vascular chambers are based on the AV loop technique principles [Colour figure can be viewed at http://wileyonlinelibrary.com]

*In vivo* prevascularization approaches effectively make use of the host body as a bioreactor for neovascularization. The main difference to *in vitro* prevascularization is that the tissue graft is implanted in the host, often in a secondary location, prior to its implantation at the primary site, that is, the site for tissue replacement. As such, this method relies on angiogenic ingrowth into the tissue‐engineered graft, which will subsequently be supplied with oxygen and nutrients (Figure 2a). Graft vascularization occurs over several weeks, depending on graft size, after which it will be surgically removed and inserted into the target site. A connection between the host and graft vasculature may be established through surgical means or through natural inosculation, but surgical anastomosis results in a more instantaneous perfusion. An often mentioned disadvantage of this technique is the multiple surgeries required, as well as the possible necessity of reseeding prior to the final implantation due to tissue necrosis caused by nutrient and oxygen deprivation at the initial implantation site (Laschke et al., 2009). Nevertheless, these *in vivo* vascularization approaches are promising as there have already been examples of successful clinical application, such as augmenting the angiogenic response, implantation of a pre‐existing artery into the tissue graft prior to graft implantation (Koepple, Kneser, & Schmidt, 2017), or prevascularization at a secondary site, for example, using the flap technique or the arteriovenous (AV) loop technique (Eweida et al., 2015; Kokemueller et al., 2010; Polykandriotis et al., 2007; Weigand et al., 2016).

### 3.4 Augmented angiogenesis

A seemingly straightforward approach to prevascularization is to augment the naturally occurring angiogenic response and thereby reduce the avascular period the tissue graft experiences (Figure 2b). The local micro‐environment of cells within a tissue or tissue graft can be supplemented with angiogenic growth factors that are important for stimulating the proliferation, differentiation, and migration of endothelial cells. Basic fibroblast growth factors are potent mitogens for mesodermal and ectodermal cells and have been shown to stimulate migration and invasion by endothelial cells *in vitro*, resulting in the formation of capillaries *in vivo* (Montesano, Vassalli, Baird, Guillemin, & Orci, 1986; Risau, 1990). VEGF is a heparin‐binding growth factor that targets endothelial cells and is thus a major contributor to the angiogenic response (Basagiannis et al., 2016). Beyond that, various other growth factors that have an effect on neoangiogenesis, both directly and indirectly, and their application in tissue constructs is a strategy to induce neoangiogenesis *in vitro* (Nomi, Miyake, Sugita, Fujisawa, & Soker, 2006). Prevascularization techniques have included supplementation of angiogenic factors to enhance vascularization. Several different approaches for delivering angiogenic growth factors have been described, with the most promising including slow‐releasing biomaterials, inducing cells to overexpress angiogenic factors through gene transfer/editing, and immobilization of proteins on scaffolds.

One challenge in the delivery of angiogenic factors is their instability *in vivo*. For example, the half‐life of VEGF under normoxia is approximately 30–45 min (Shima, Deutsch, & D'Amore, 1995). This makes direct intravenous delivery (bolus administration) insufficient for providing sustained local VEGF at the right concentrations, a requirement for establishing a mature vascular system (Des Rieux, Ucakar, Mupendwa, et al., 2011). Importantly, as VEGF also regulates vascular permeability, its overload may cause leaky vasculature and hypotension, making controlled release crucial (Hariawala et al., 1996). Therefore, in an effort to find a more effective method of providing angiogenic factors to a tissue graft in a local, long‐term manner, biomaterials have been designed to consistently and gradually release growth factors (Figure 2b, left panel).

Hydrogels and porous polymeric scaffolds are two types of biomaterials that allow cell incorporation and subsequent vascularization, while also being capable of being loaded with growth factors (Holland et al., 2007; Peters, Polverini, & Mooney, 2002; Sheridan, Shea, Peters, & Mooney, 2000). There are various methods of loading growth factors, such as direct loading, covalent binding, or making use of electrostatic interactions (Silva, Richard, Bessodes, Scherman, & Merten, 2009). Another variation of using biomaterials for growth factor delivery is delivering growth factors bound to water‐soluble substances such as polyelectrolytes, thereby extending their half‐life. One example hereof is the use of the heparin‐binding domain of VEGF to bind polyanion dextran sulphate, which enabled a sustained release of VEGF that persisted for more than 10 days (Huang, Vitharana, Peek, Coop, & Berkland, 2007). When using this encapsulation technique in combination with hydrogels and poly(‐lactic‐*co*‐glycolic acid) scaffolds, it was shown that release of VEGF was mostly affected by this encapsulation, likely because of increased protection from hydrolytic degradation (des Rieux et al., 2011).

A further improvement on this technique was made with a fibrin hydrogel system containing ionic‐albumin microspheres, which allowed for time‐controlled release of two rather than one growth factors, resulting in vascularization 8 weeks after implantation of the tissue graft (Layman et al., 2012). Although these techniques have shown significant improvements in vascularization of tissue grafts (Layman et al., 2012; Peters et al., 2002), there have been problems with low protein loading and loss of biological activity of the growth factors upon their release from the scaffold or capsule (Golub, Kim, Duvall, et al., 2010; Sheridan et al., 2000).

Another, more classical, approach to improving angiogenesis is the immobilization of proteins and growth factors on biomaterials. For example, cell adhesion peptides are used to enhance the adhesion and migration of endothelial cells, for example, by coating biomaterials with ECM molecules, thereby improving angiogenesis. Short peptide sequences such as RGD, REDV, or SVVYGLR have also been shown to improve cell adhesion and improve vascularization compared with non‐modified substrates (García, Clark, & García, 2016; Hamada et al., 2003; Wang et al., 2015). One of the most promising aspects of this approach is the possibility to selectively recruit cells. However, although this technique has proven successful in enhancing the angiogenic response, it alone is not capable of ensuring adequate neovascularization.

An alternative approach to supplying sufficient growth factors is using cells that overexpress angiogenic factors due to gene transfer or gene editing, allowing for continuous growth factor release (Figure 2b, right panel). Gene transfer or editing has been accomplished mainly through viral vectors although new nonviral methods have become a focus of current research in an effort to improve safety. The basic principle is transfection or transduction of growth factor genes into cells of the tissue graft. Using viral vectors, these can be immobilized on the surface of the implanted material (gene‐activated matrixes, allowing for DNA uptake by invading cells *in vivo*, resulting in improved vascularization (Fang et al., 1996; Shea, Smiley, Bonadio, & Mooney, 1999). Nonviral approaches include the use of ultrasound‐targeted microbubble destruction, gene transfer via electroporation, or through mineral‐coated hydroxyapatite microparticles, which have all shown capable of transferring growth factor genes that have a positive effect on angiogenesis (Bucher, Gazdhar, Benneker, Geiser, & Gantenbein‐Ritter, 2013; McMillan et al., 2018; Shimoda, Chen, Noguchi, Matsumoto, & Grayburn, 2010). Although nonviral vector transduction has been less well‐established and requires further research to determine its clinical applicability, its development shows an improvement over viral methods, as there is no chance of direct exposure to viral vectors. Moreover, new methodologies like the CRISPR/Cas9 system hold promise, as they could be used to, for example, inhibit angiostatic pathways, as such enhancing angiogenesis, and their efficacy in a clinical setting will most likely be shown soon.

### 3.5 Prevascularization at a secondary implantation site

As an alternative, prevascularizing a tissue graft *in vivo* at a secondary site of implantation could assist in providing adequate vascularization. One of these approaches is the flap technique (Figure 2c). The flap technique builds upon the idea of natural angiogenic ingrowth by implanting the tissue construct into a muscle flap, which is subsequently transferred to the target site together with the tissue graft. Of interest is a study that used the flap technique to achieve growth and *in vivo* prevascularization of bone grafts (Kokemueller et al., 2010). In a sheep model, the latissimus dorsi muscle was used as the site of initial implantation of osteogenic material allowing for bone and highly vascularized fibrous tissue development in the transplanted cylinder containers, which were still present 3 months post‐implantation, demonstrating the feasibility of this technique. This study also managed to apply this technique in a human patient to replace the left hemimandible, where vascularized bone grafts were prefabricated over 6 months. After the final surgery, there were no complications and a follow‐up after 2 months showed no indications of graft rejection. This study thus showed the feasibility of the flap technique in both an experimental setting and a clinical application, in which adequate prevascularization could provide a clinically relevant long‐term solution. An additional advantage of the flap technique is the possibility for patient‐specific reconstruction by shaping the container that holds the tissue graft to match a 3D reconstruction of the patient's tissue, for example, a piece of skull. Nevertheless, the technique still imposes donor site defects as parts of the muscle need to be transplanted together with the tissue graft to ensure instantaneous perfusion, a shortcoming that needs to be considered (Warnke et al., 2004).

An advancement on the flap technique that warrants mentioning is the AV loop technique for which the first steps were made by Erol and Spira (1979) when they demonstrated an angiogenic response resulting from an AV fistula shaped like a loop, most likely due to angiogenic factor release and local shear stress. The AV technique is an improvement on the flap technique because it may be transferred from the site of *in vivo* prevascularization to the target site without the loss of donor tissue at the secondary site of transplantation (Figure 2d). This is due to the intrinsic properties of the prefabricated AV loop technique that does not require embedding of the tissue graft into the surrounding tissue. In contrast, the tissue construct is contained within a growth chamber that is placed around the AV loop, which is only connected to the host's vascular system (Eweida et al., 2015; Polykandriotis et al., 2007; Weigand et al., 2016). This growth chamber, usually made from polycarbonate, may be either empty or supplemented with ECM components. It can be shaped however needed, and it may be implanted at almost any site allowing for a high degree of flexibility in a clinical setting (Mian et al., 2000). Various studies have shown successful vascularization of skin, cardiac, adipose, cartilage, and bone tissue, whereas the flap technique is mostly limited to bone grafts (Boos et al., 2013; Burghartz et al., 2015; Kneser et al., 2006; Messina et al., 2005; Morritt et al., 2007; Tanaka, Tsutsumi, Crowe, Tajima, & Morrison, 2000). Additionally, the AV loop technique has, similar to the flap technique, been used to generate large vascularized bone grafts. Importantly, *de novo* bone formation was achieved without the creation of significant donor site defects (Horch, Beier, Kneser, & Arkudas, 2014), showing a clear advantage over the flap technique.

Although tissue grafts in an AV loop environment do experience hypoxia, the peak value of hypoxia coincided with the peak of cell proliferation at 7 days post‐implantation (Hu et al., 2008). This may be due to the positive effect hypoxia has on the generation of angiogenic factors such as VEGF or may reflect the increased local metabolic demand due to the high proliferation rate. Furthermore, a period of increased angiogenesis was observed, which occurred after the peak of hypoxia between 7 and 10 days post‐implantation, supporting a potential local increase of angiogenic factors (Lokmic, Stillaert, Morrison, Thompson, & Mitchell, 2007). This angiogenic response can additionally be enhanced by encapsulation of MSCs (Steiner et al., 2018). Moreover, adaptations of the AV loop technique, for example, vascularized chambers that use the encapsulation of an artery and vein instead of using a vein graft‐based AV loop, have also been developed and have been reviewed recently (Yap, Yeoh, Morrison, & Mitchell, 2018).

Overall, a combination of the aforementioned approaches to augment angiogenic responses may very well be integrated with *in vitro* or *in vivo* prevascularized tissue constructs, as this may be more effective than using them as a standalone technique.

## 4 MICROFLUIDIC AND ENGINEERING APPROACHES TO VASCULARIZATION IN TISSUE ENGINEERING

Although the biology‐based approaches focus on using and enhancing or augmenting natural blood vessel formation, an alternate approach focuses on providing an engineered basis for the growth and establishment of blood vessels. This engineering‐centric approach is based on the synthetic construction of tubular scaffolds. As described, scaffold properties and design can have a significant impact on the successful enhancement of vascularization, and tubular scaffolds are an alternative approach to aid in the immediate vascularization of a tissue construct. Scaffolds may be constructed to control growth factor release (Layman et al., 2012) or have a direct impact on endothelial cells. Application of electrospun scaffolds, for example, allows for tailored fibre diameter and orientation. Optimizing fibre orientation has been shown to increase cell alignment, which in turn improves endothelial cell attachment as cells that have higher degree of alignment have more vinculin expression. As a result, the cells can withstand higher flow rates and are better able to resist deformation or detachment, a crucial aspect in ensuring the correct formation of blood vessels (Whited & Rylander, 2014). Taking this idea a step further, several techniques focus on manufacturing a physical blueprint for a vascular network and vascular patterning, as has been more extensively reviewed recently (Malheiro, Wieringa, Mota, Baker, & Moroni, 2016).

### 4.1 Microfluidic scaffolds

One such technique is the use of microfluidic scaffolds, which aim to form artificial capillary networks through various moulding processes within a biomaterial. Importantly, these channels can be endothelialized, resulting in vascularized tissue constructs that allow for adequate oxygen and nutrient supply. As vascularization is dependent on the presence of flow, creating a microfluidic scaffold that can apply a local flow over the endothelial cells represents an additional approach to enhance tissue graft vascularization.

In early approaches, nonbiodegradable materials such as silicon and Pyrex were used in combination with photolithographic techniques to provide a template scaffold for cells. Subsequently, the monolayers obtained from these templates were lifted and folded into vascularized 3D tissue constructs (Kaihara et al., 2000). Polydimethylsiloxane (PDMS) has been frequently used for replica moulding (Duffy, McDonald, Schueller, et al., 1998; Leclerc, Sakai, & Fujii, 2003; Rosano et al., 2009; Shin et al., 2004) due to its low chemical reactivity and non‐toxicity towards endothelial cells for a duration of up to 4 weeks (Borenstein et al., 2002). Furthermore, PDMS can be cross‐linked allowing for improved control over orientation and introducing the possibility to establish various chemical functionalities on microchannels (Abdallah & Ros, 2013). PDMS, however, is hydrophobic, resulting in unpredictable absorbance of molecules in the channels (Borenstein et al., 2010; Hu et al., 2004). Also, the nondegradability of these materials imposes major limitations for clinical applications as the implantation of a nonbiodegradable scaffold could require an additional surgery for its removal, increasing the risk to the patient (Patel & Fisher, 2008).

To overcome this problem, biodegradable materials have also been investigated. Poly(glycerol sebacate) (PGS) implants, for instance, are completely absorbed within 60 days after subcutaneous transplantation in animal models, thus providing a marked improvement for the patient (Wang, Ameer, Sheppard, & Langer, 2002). Additionally, endothelial cells and other (tissue‐specific) cell types are able to adhere to and proliferate on PGS surfaces. In one study, replica moulding was used to create a scaffold out of PGS with tubular channels, which was subsequently endothelialized, reaching confluency after 2 weeks of culture (Fidkowski et al., 2005). Hydrogels are also often employed for creating microfluidic channels due to their ECM‐like properties, thus resulting in better biocompatibility and biodegradability (Ling et al., 2007; Slaughter, Khurshid, Fisher, Khademhosseini, & Peppas, 2009). Importantly, these studies demonstrated that it is not only the material properties but also the microfluidic techniques used that influence the viability of these tubular scaffolds for tissue engineering and regenerative medicine.

**Figure 3:**
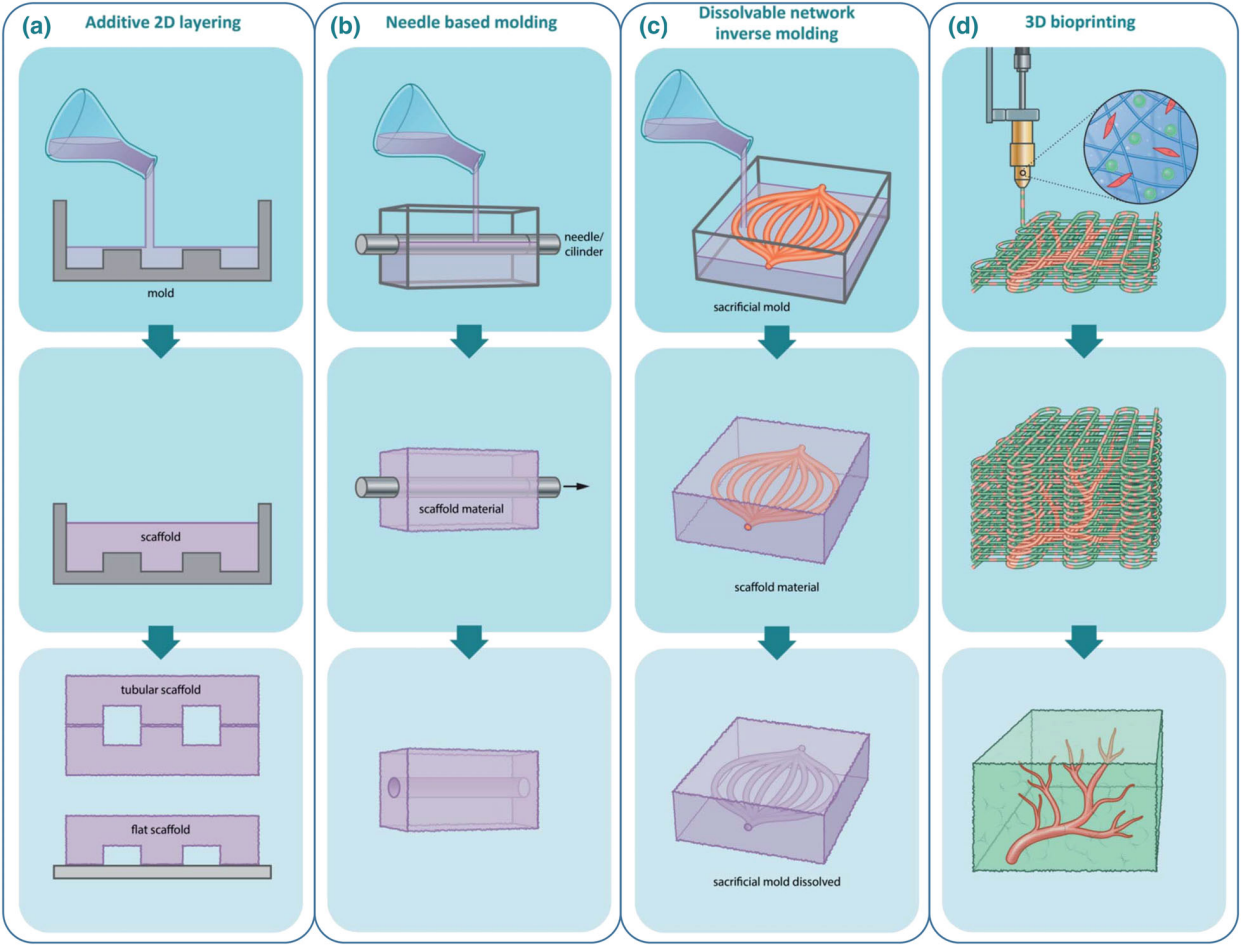
Engineering‐based approaches to creating vasculature and vascularized grafts. Beyond the biology‐inspired approaches, engineering‐based approaches have also been developed to achieve vascularization. (a) Additive layering has been used to build tubular structures to build blood vessels. In this technique, scaffolds are made by moulding, after which they can be stacked to either tubular or flat channels. A limitation of this technique is that the channels usually have square or rectangular morphologies, which are unfavourable for cells. (b) A similar technique that can be used to create round channels is needle‐based moulding, mimicking the morphology of vessels *in vivo*. (c) A derivative of this technique, which allows the creation of more complex vascular structures, is inverse moulding using a sacrificial mould. The sacrificial mould is embedded in the scaffold material, after which it is dissolved, leaving of tubular structure that in turn can be vascularized and integrated with a tissue graft. (d) Most promising may be the combination of biology‐ and engineering‐based approaches, for example, 3D bioprinting. Here, it is possible to combine printing of different cell types in 3D to build a cellularized graft with pre‐existing vessels, which should, upon implantation, yield a graft that does not suffer from hypoxia or nutrient deprivation [Colour figure can be viewed at http://wileyonlinelibrary.com]

In terms of microfluidic techniques, several processes have been described, the first of which is the moulding process. This moulding processes may be separated into additive and subtractive methods, where additive methods mostly rely on the stacking of two‐dimensional scaffolds containing moulds that form a channel when stacked, whereas subtractive methods make use of cylindrical templates that can be removed from the scaffold, thereby creating empty tubular networks (Hasan et al., 2014). In the additive method, the formation of the mould plays an integral role. In replica moulding, photolithography or soft lithography is most often used to create the master mould with the necessary structural features (Figure 3a). These structural features are transferred to the transfer mould by pouring the scaffold material onto the master mould. This scaffold may then be allowed to adhere on either a flat scaffold or another transfer mould to construct tubular scaffolds (Borenstein et al., 2002; Fidkowski et al., 2005).

Subtractive methods may be further separated into two main categories: needle‐based moulding methods (Figure 3b) and dissolvable network‐based sacrificial moulding (Figure 3c). Needle‐based moulding methods provide a relatively simple approach for obtaining vascularized scaffolds, where a needle (or another cylindrical structure) is inserted into the scaffolding material and removed after cross‐linking (Chrobak, Potter, & Tien, 2006). Dissolvable network‐based sacrificial moulding is based on the formation of an inverse mould with an easily dissolved material, which is then encased in a 3D gel scaffold. The inverse mould is dissolved and as such removed from the 3D scaffold, leaving a fully interconnected, vascularized 3D scaffold (Golden & Tien, 2007; Lee et al., 2010). This technique has the advantage of being highly versatile as both the network branching and the capillaries' individual width may be controlled.

A major restriction of these microfluidic techniques is the geometry of the microfluidic channels, as replica moulding usually results in a rectangular cross section rather than the physiological rounded vessels. As a result, channel intersections usually contain sharp corners and abrupt width changes, which negatively impact cell seeding, thus hindering the development of fully endothelialized blood vessels (Borenstein et al., 2010; Green et al., 2009). Furthermore, many studies were unable to produce sufficiently small channels (Chrobak et al., 2006; Fidkowski et al., 2005). As the majority of a tissue section is provided access to nutrients and oxygen via capillaries as small as 3–4 μm in diameter, achieving these values is crucial for the engineering approaches to avoid hypoxic conditions in the tissue graft that could lead to tissue necrosis. One possibility for overcoming these limitations would be the construction of microvascular networks built with circular cross sections, for example, by using microthermoforming techniques as currently applied in *in vitro* models (Hebeiss, Truckenmuller, Giselbrecht, et al., 2012; Truckenmuller, Giselbrecht, van Blitterswijk, et al., 2008), where round channels with smooth transitions better mimic the natural physiological micro‐environment. One obstacle is the precise alignment of the two half round channel surfaces. Moreover, the compaction and contractility of the substrate are important for, for example, inducing VEGF gradients and VEGFR2 expression, showing that substrate characteristics should be considered carefully (Rivron et al., 2012).

### 4.2 3D (bio)printed scaffolds

Considering these limitations, 3D printing is a very promising alternative, as it seems to provide the means to create rounded channels, and it displays great versatility in terms of successfully engineering larger vascularized grafts. 3D printing techniques allow for better cell regulation by controlling their location and distribution of proteins and other chemical cues in the micro‐environment. Direct ink writing, for example, allows for controlling the pattern of the scaffold in three dimensions as fugitive organic ink is structured into vascular networks prior to encapsulation and subsequent removal (Fu, Saiz, & Tomsia, 2011; Lewis, 2018). Further improvements are presented by omnidirectional printing, which removes the need for layer‐wise patterning, as the fugitive organic ink is printed into a photopolymerizable gel allowing for even greater versatility in achieving physiologically suitable vascularization (Wu, DeConinck, & Lewis, 2011). Equally important is the possibility to include cells in these scaffolds. 3D bioprinting (Figure 3d) presents an attractive opportunity for this, as it allows coprinting of a vascular blueprint, cells, and ECM components, resulting in heterogeneous tissue constructs (Kolesky, Truby, Gladman, et al., 2018). Furthermore, the implantation of 3D printed vascularized grafts has shown spontaneous but structurally ordered angiogenesis *in vivo*. A study found that these vascularized grafts improved perfusion of tissues, thereby preventing further tissue necrosis, demonstrating the feasibility of applying this technique in a clinical setting (Mirabella, MacArthur, Cheng, et al., 2017). Although to this date, no clinical trials have been conducted with this technology and even though the techniques require some additional refinement, 3D bioprinting appears to be a valuable and potentially powerful approach for solving one of the major challenges in achieving vascularization in tissue‐engineered grafts.

## 5 CONCLUSION

In this review, we provided an overview of both classical and state‐of‐the‐art approaches for vascularizing tissue‐engineered constructs. Overall, these approaches are either biology‐based or engineering‐based, and all have their own distinct advantages and disadvantages. Stem cell research and biomaterial engineering have demonstrated both the feasibility of vascularized tissue constructs and, as a consequence, the importance of vascularization. It is becoming clear that the various strategies to achieve vascularization are making progress towards the generation of clinically relevant tissue grafts. Although both approaches have demonstrated the importance of ensuring appropriate oxygen supply, the biology‐based strategies have shown that the micro‐environment of the tissue‐specific cells is vital to ensure adequate vascularization and by extension to ensure tissue growth without a necrotic core. The significant influence that ECM molecules, growth factors, angiogenic factors, and their release in a physiological manner have on tissue growth further supports this notion. Significantly, engineering‐based approaches also reinforce the importance of the micro‐environment. Electrospun topographies have been shown to have an impact on alignment and adhesion properties of endothelial cells, and the biomaterials used for scaffolds can have an equally significant impact in terms of proliferation and differentiation, thus affecting clinical application.

Although the tissue engineering and regenerative medicine field has taken great steps in pursuing various avenues to obtain clinically relevant tissue‐engineered grafts with an adequate and functional vasculature, the combination of the various strategies will be essential. Although 3D bioprinting techniques are still being developed and improved, incorporation of, for example, growth factors and other angiogenic factors could significantly enhance graft vascularization. The biomaterials for scaffolds should be selected carefully, and together, the choice of bioprinting technique, scaffold material, and biological factors will all influence the cell fate of both endothelial and tissue‐specific (stem) cells. Applying this in combination with prevascularization approaches is most promising to ensure tissue graft survival, taking into account the importance of flow in establishing a functional vascular bed within the tissue graft. The graft will be better adapted to instantaneous perfusion, which in turn establishes a continued nutrient and oxygen delivery to the cells. Beyond this, newly emerging approaches, such as vascular organoids (Wimmer et al., 2019), may not only help us study vascularization in graft models but also potentially help establish a better graft vasculature. In conclusion, efforts should be made to integrate the currently available approaches by combining their strengths and eliminating their weaknesses.

## CONFLICT OF INTEREST

The authors have declared that there is no conflict of interest.
